# Cholecystogastric fistula in Bouveret syndrome: Case report and literature review

**DOI:** 10.1016/j.ijscr.2022.106918

**Published:** 2022-03-10

**Authors:** Carlos Eduardo Rey Chaves, Carlos Jose Villamil, Saralia Ruiz, Valentina Galvis, Danny Conde, Juan Carlos Sabogal Olarte

**Affiliations:** a Faculty of Medicine, Universidad del Rosario, Colombia; b Hospital Universitario Mayor Méderi, Colombia

**Keywords:** Bouveret syndrome, Gallstone ileus, Obstruction, Endoscopy, Surgery

## Abstract

**Introduction and importance:**

Cholelithiasis is the benign bile pathology with major prevalence. A rare condition has been described, when a stone migrates through the duodenum causing small bowel obstruction (SBO), it's known as Bouveret syndrome, and it's attributed to almost 5% of SBO. Just 2% of the cases present with the migration of the stone through a fistula between gastric chamber and gallbladder, with limited reports in the literature.

**Clinical findings:**

We present a case of an 87-year-old male with Bouveret syndrome and a cholecystogastric fistula with a stone in the gastric chamber who underwent laparoscopic gastrotomy to resolve the clinical case.

**Conclusion:**

Bouveret syndrome remains to be a rare condition in benign bile pathology. Individualized treatment should be performed and multidisciplinary approach leads to improved outcomes for the patient.

## Highlights

• Bouveret’s syndrome it’s a rare entity causing approximately 5% of small bowel obstruction.
• Multidisciplinary approach leads to good postoperative outcomes
• Endoscopy it’s the best approach to resolve bile stones, however, laparoscopic approach needs to be in the armamentarium

## 1 Introduction and importance

Benign biliary disease is one of the main reasons for admission to the emergency department [1]. The pathology with the highest prevalence to which 95% of cases are attributed is cholelithiasis [1]. There are very rare cases where a gallstone migrates to the duodenum through a cholecystoduodenal fistula causing proximal obstruction, this pathology is known as Bouveret syndrome and less than 5% of cases of intestinal ileus due to obstruction are attributed to it [1], [2], [3]. In addition to being an uncommon pathology and having few reports in the literature in recent years, it is even more atypical for this syndrome to occur with the migration of the stone through an abnormal path between the gallbladder and the stomach generating an intermittent pyloric obstruction with nonspecific clinical signs (approximately 2% of what has been reported in the world wide literature) [1], [2], [3], [4], [5], [6] The objective of this article is to present a clinical case of Bouveret syndrome presented with pyloric obstruction due to cholecystogastric fistula.

## 2 Presentation of the case

**Fig. 1:**
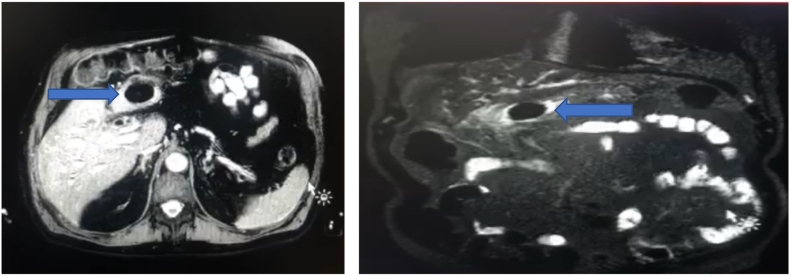
Left: MRI. Stone in gastric chamber. Right: MRI. Gall bladder adhesion to gastric chamber.

After ethical and institutional approval, previous informed consent filled, following SCARE guidelines [7]. An 87 year old male patient was admitted to the emergency department due to 1 month of abdominal pain associated with unquantified febrile peaks, chills, multiple emetic episodes, nausea, asthenia and adinamia. Surgical history of open radical prostatectomy 10 ago due to benign prostatic hyperplasia. On physical examination at the moment of admission, the patient presented mild abdominal pain to deep palpation in the right hypochondrium, without any signs of peritoneal irritation. Laboratory studies were requested in which leukocytosis of 11,000 was evident, liver function tests altered by hypertransaminasemia, alkaline phosphatase at 998 IU/L and hyperbilirubinemia at the expense of direct at 2.44 mg/dL. Additionally, the patient had extra institutional ultrasonography in which a space-occupying lesion related to the liver and cholelithiasis was evident. An abdominal MRI was performed which showed an abnormal path between the vesicular fundus and the pylorus, with the passage of the stone into the stomach. The stone was in the antropyloric region, associated with chronic inflammatory changes in the gallbladder, pneumobilia and dilation of the bile duct without choledocholithiasis (Fig. 1).

Upper digestive tract endoscopy was performed, documenting the presence of a large pigmented calculus of more than 2 cm lodged in the fundus and proximal body along with the distal antrum towards the greater curvature with a deep fistulous orifice approximately 12 mm, subsequently, an endoscopic retrograde cholangiopancreatography was requested, which confirmed the presence of the stone, during the procedure there was a failed attempt to remove the calculus with a conventional basket. A second failed attempt was made in which a polyps extraction mesh was used, even though, this does not manage to pass through the esophagus, so a basket of lithotripsy was used looking to generate the appropriate pressure to break the calculus, however, it was not possible to break the stone. Additionally during the procedure it was possible to observe a scar in the duodenal bulb towards the anterior semicircle, but no holes or fistulas were observed.

**Fig. 2:**
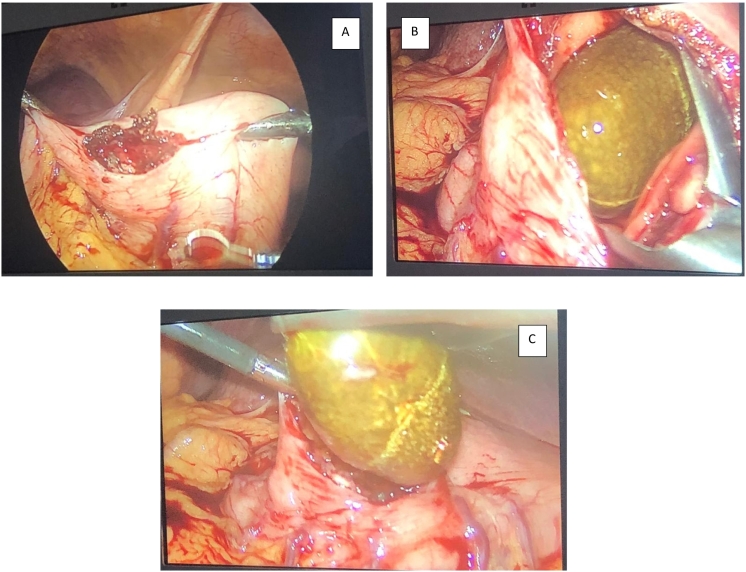
A: Laparoscopic gastrotomy. B: Direct visualization of the stone. C: Stone extraction.

Finally, after the failed attempts, the decision to take the patient to surgical extraction of the stone by laparoscopic approach was made, in which gastrotomy was performed along the greater curvature, the calculus was removed with an extraction clamp, and a primary raffia of the stomach was made by an Hepatobiliary and pancreatic surgeon (Fig. 2). Due to the severe inflammatory process evidenced in the gallbladder which conditions the presence of a phlegmon, it was decided to defer surgical management. The procedure was carried out without complications. The patient had a hospital stay of 2 days, where the adequate oral tolerance was verified. After 30, 60 and 90 days, no associated complications were observed.

## 3 Discussion

This syndrome was first described in 1770 by Beaussier, however it was named after the French physician Leon Bouveret after he published two cases of this syndrome more than 100 years later in 1896 [1], [2], [3], [4], [5], [7]. Bouveret syndrome is a rare complication of cholelithiasis, more precisely, it is a very rare variant of biliary ileus. Biliary ileus is a well-known complication of cholelithiasis that usually occurs after recurrent episodes of cholecystitis due to an important inflammatory process that generates adhesions between the gallbladder and the digestive tract [7], [8], [9], [10]. It presents as an intestinal obstruction caused by the migration of a large gallstone by means of a cholecystoenteric fistula. The stone travels through the intestinal tract until it reaches the ileocecal valve where due to its large size, it cannot manage to pass into the colon and an intestinal obstruction occurs. The most common obstruction documented occurs in the terminal ileus (60–70%) followed by the proximal ileum (25%), distal ileum (10%), jejunum (9%), colon (4%), rectum (2%) and duodenum as the rarest site of obstruction reaching 1% to 3% [1], [2], [3]. On the other hand, in Bouveret syndrome, the migration of the gallstone, despite also being generated by a fistula, has not the same characteristics because it occurs more commonly as a cholecystoduodenal fistula. In those patients who present this complication it is common to see that obstruction of gastric emptying develops because, unlike the cases of biliary ileus known, the stone generates an obstruction at the proximal portion of the duodenum and even in the pylorus instead of further distally locations [6], [7], [8], [9], [10]. Risk factors include the female sex, age over 70 years, recurrence in inflammation of the gallbladder leading to erosion and necrosis of the walls favoring the formation of bilioenteric fistulas and the presence of stones >2.5 cm has been related to proximal obstructions [10], [11], [12]. Clinical presentation and symptoms vary depending on the site of the obstruction. The onset of symptoms can be acute, subacute or chronic, the spectrum of symptoms include abdominal pain, nausea, vomiting, and dyspepsia in case of gastric emptying obstruction [7], [8], [9], [10], [11], [12]. In terms of diagnostic images, the Rigler's Triad can be identified in 33% of cases with conventional abdominal radiography. However, the use of CT allows identifying more clearly the pneumobilia, distension of abdominal loops and the embedded stone in the ileocecal valve, adding the possibility of determining fistulous paths, the degree of obstruction and inflammation and the location of the stones with a sensitivity of 93% and specificity of 100%. Abdominal ultrasound is useful in 60% of cases finding signs of cholecystitis, abdominal distension and pneumobilia [1], [2], [3], [10], [11], [12], [13], [14], [15].

Despite advances in management strategies, the morbidity and mortality of Bouveret syndrome has remained high, reaching 60% morbidity and 30% mortality. This is attributed principally to the pre-existence of comorbidities of affected patients and the fact that the symptoms are nonspecific, which makes timely diagnosis difficult [10].

Treatment of Bouveret syndrome can be both endoscopic and surgical [3], [4], [5], [16], [17], [18]. There are multiple modalities for the treatment of Bouveret syndrome. Among these, there are endoscopic approaches that include endoscopic extraction itself, endoscopic laser lithotripsy, extracorporeal shock wave lithotripsy and intracorporeal electrohydraulic lithotripsy [1], [2], [3]. The endoscopic approach, first successfully described by Bedogni in 1985 [4], has been shown to have good results in the extraction of stones in the proximal intestine with good results in high-risk patients. In 2001 Lambrenghi et al. [5] also describes an unusual localization of the gallstone in Treitz ligament, treated successfully with endoscopic approach. However, the endoscopic approach beyond being an operator dependent procedure and requiring specialized tools, is limited to smaller calculus, usually less than 3 cm. Park, et al. [16], [17], [18] present that up to 91% of endoscopic and percutaneous extraction attempts fail, requiring subsequent surgical approach, as happened in our case, despite having a specialized team in endoscopic extraction technique [16], [17], [18]. On the other hand, the surgical approach includes laparoscopy and laparotomy techniques with gastrotomy, pylorotomy, duodenotomy in those cases in which the mobilization of the stone is easy and there is no excessive ulceration of the mucosa [2].The performance of cholecystectomy and fistula repair have been also described, however given the increase in morbidity and mortality in older patients it is recommended to carry out two differed surgical procedures [19], [20], [21]. In the first instance resolve the obstruction by removing the stone and then perform cholecystectomy and repair of the fistula. Although open surgery procedures have been linked to increased morbidity and mortality, Al-Habbal et al. [22], [23], [24], [25], presented a series of 161 case reports of which, 146 (71%) were managed by laparotomy and extraction of the stone by enterotomy or gastrostomy with successful results on endoscopic and laparoscopic approach.

The case presented is unique considering the presence of a cholecystogastric fistula, which is an extremely rare presentation within the natural history of this complication. Additionally, the location and size of the stone hindered the initial endoscopic management and when performing laparoscopic approach through gastrostomy along the greater curvature, fistula repair of the fistulous path was not performed due to self-resolution of it. Similarly, it was decided to evaluate the performance of cholecystectomy in a second surgical time since statistically due to the age of the patient there would be a greater risk of postoperative morbidity and mortality [10], [11], [12], [19], [20], [21].

## 4 Conclusion

In conclusion with the current scientific evidence and reported cases of Bouveret syndrome, the management of this rare condition is subject to the individual characteristics of each patient considering factors such as age, nutritional status and comorbidities. It is of great importance to evaluate the risk of the different approaches and the performance of surgery in one or two different times to provide the patient with the treatment option with the highest probability of success, lower morbidity and mortality and decrease in hospital stay.

## Provenance and peer review

Not commissioned, externally peer-reviewed.

## Consent

Written informed consent was obtained from the patient for publication of this case report and accompanying images. A copy of the written consent is available for review by the Editor-in-Chief of this journal on request.

## Ethical approval

Ethical approval of institutional committee was made previous publication.

## Funding

This research did not receive any specific grant from funding agencies in the public, commercial, or not-for-profit sectors.

## Guarantor

Carlos Rey.

## Research registration number

None.

## CRediT authorship contribution statement

**Carlos Rey, MD:** Participate in drafting the article and revising it critically for important intellectual content.
**Carlos Villamil:** Make substantial contributions to conception and design, acquisition of data, analysis and interpretation of data.
**Valentina Galvis:** Participate in drafting the article and revising it critically for important intellectual content.
**Saralia Ruiz:** Participate in drafting the article and revising it critically for important intellectual content.
**Danny Conde, MD, Msc:** Participate in drafting the article and revising it critically for important intellectual content.
**JC Sabogal, MD, Msc:** Give final approval of the version to be submitted and any revised version.

## Declaration of competing interest

Authors do not declare any conflict of interest.
